# Trefoil factor 3 contributes to the malignancy of glioma via regulating HIF-1α

**DOI:** 10.18632/oncotarget.20010

**Published:** 2017-08-07

**Authors:** Shuo Diao, Qianqian Zheng, Jian Gao, Yiqun Yao, Siyang Ren, Yongjian Liu, Yinghui Xu

**Affiliations:** ^1^ Department of Neurosurgery, First Affiliated Hospital, Dalian Medical University, Dalian, People's Republic of China; ^2^ Department of Pathophysiology, Basic Medical College, China Medical University, Shenyang, People's Republic of China; ^3^ Center of Laboratory Technology and Experimental Medicine, China Medical University, Shenyang, People's Republic of China; ^4^ Department of Interventional Therapy, First Affiliated Hospital, Dalian Medical University, Dalian, People's Republic of China

**Keywords:** TFF3, glioblastoma, HIF-1α, proliferation, apoptosis

## Abstract

Trefoil factor 3 (TFF3) plays significant roles in several solid tumors. However, the expression pattern and function of TFF3 in glioblastoma (GBM) have not been reported. Here, we report that expression level of TFF3 significantly elevated in glioma and correlated with the prognosis of glioma patients. Then we found TFF3 promotes proliferation, invasion, and migration and inhibits apoptosis of glioma cells *in vitro*, and delayed tumor progression in subcutaneous xenograft nude mice, and prolonged the median survival time in orthotopic xenograft mice. Moreover, knockdown of TFF3 reduced the expression of HIF-1α through a hypoxia-independent manner. These findings suggest that targeting TFF3 may offer a novel strategy for therapeutic intervention of malignant gliomas.

## INTRODUCTION

Glioblastoma multiforme (GBM), one of the most common types of primary brain tumors, is regarded as the most common and malignant type of tumor of the central nervous system in adults [1, 2]. Clinical treatment is less efficient due to the increased invasion, migration, proliferation and decreased apoptosis of these tumors cells. Thus, research on these molecular and cellular mechanisms could deepen our understanding of gliomas, as well as provide effective targets and valuable insights to improve patient outcomes.

Trefoil factor 3 (TFF3), also known as intestinal trefoil factor (ITF), is predominantly expressed in the intestinal goblet cells [3, 4]. TFF3 plays a key role in mucosal integrity and repair [5, 6]. In the epithelial tissue, TFF3 acts as a mitogenic factor in epithelial restitution after wounding or during inflammation [7]. Additionally, TFF3 is expressed in normal lung, colon, stomach, pancreas, spleen and liver, among other organs [8]. Recent studies have also disclosed that TFF3 is overexpressed in various solid carcinomas [9–12] and plays a significant role in oncogenic cell growth, angiogenesis, invasion and metastasis [13–15]. In the central nervous system (CNS), TFF3 is widely expressed in several subtype cells including oxytocinergic neurons in the hypothalamus, neurons and microglial cells in cerebral cortex [16]. Notably, TFF3 highly expressed in hippocampus [17–19], which is important in regulating learning and memory as well as mood disorders [20, 21]. The wide neural distribution of TFF3 suggests its potential roles in the CNS and related disorders.

However, the expression and biological function of TFF3 in malignant gliomas is not clear. Here, we find that TFF3 is overexpressed in glioma cell line and in glioma tissues. We also found that TFF3 promotes cell proliferation, cell metastasis and inhibits cell apoptosis by regulating Hypoxia-Inducible Factor -1α (HIF-1α) under normoxic conditions in glioma cell *in vitro*. HIF-1 is a primary oxygen-sensitive transcriptional activator and helps cells to adapt to hypoxia [22]. HIF-1 is composed of a constitutively expressed β-subunit and a hypoxia-inducible α-subunit. HIF-1α regulates HIF-1 transcriptional activity and activates multiple target genes that involve in crucial aspects of cancer progression, including angiogenesis, glucose metabolism, cell proliferation/ survival and apoptosis [23]. The aim of this study was to identify the role of which TFF3 plays as a potential oncoprotein that influences various aspects of glioma cell maneuver via regulating HIF-1α.

## RESULTS

### TFF3 expression level is increased in human glioma tissues and serves as an indicator of poor clinical outcome

**Figure 1:**
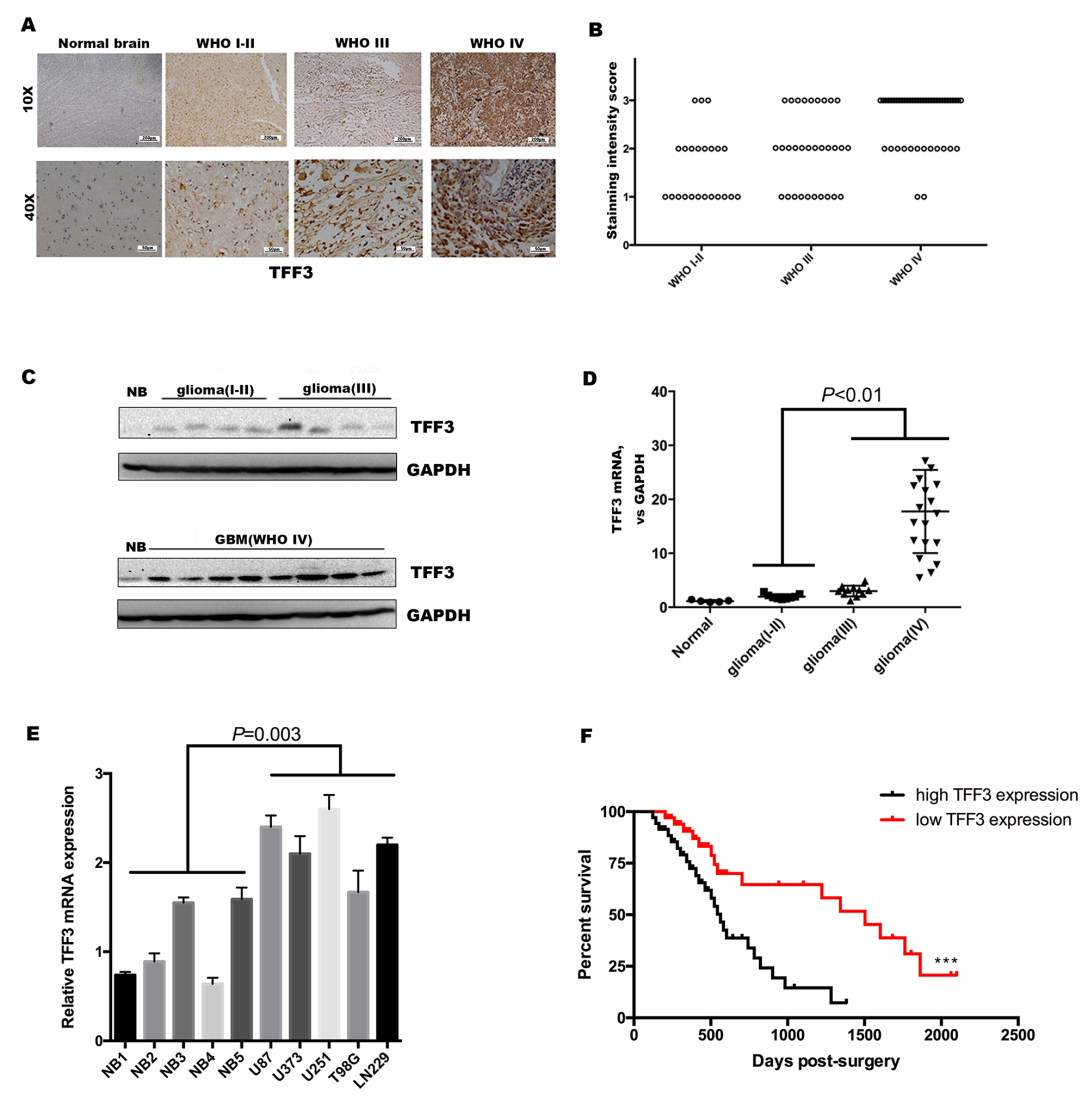
TFF3 is overexpression in glioma and serves as an indicator of poor clinical outcome **(A)** Immunohistochemistry with anti-TFF3 antibody on human glioma tissue samples showing both nuclear and cytoplasm positivity, one representative of each grade shown. Normal brain tissue samples showing negative staining **(B)** TFF3 expression, represented by the staining intensity from WHO grade I-II to grade IV, showing statistically significant differences between grade IV and grade I-II, grade III. (*P*<0.001, n=92, I-II=23, III=31, IV=38). **(C)** Western blotting analysis showing that the overall expression of TFF3 tended to increase along the sequence from normal brain (NB) to low-grade glioma (WHO grades I-II), and then to high-grade glioma (WHO grades III and IV). The results from two blots were normalized by NB. **(D)** qRT-PCR showing that the levels of expression of TFF3 mRNA were significantly higher high-grade glioma tissues than in low-grade glioma(*P*<0.01, n=40). **(E)** qRT-PCR examination of the relative TFF3 mRNA expression in five glioma cell lines and five normal brain tissues. Data are shown as mean+SD (n=3). **(F)** Kaplan-Meier analysis of the correlation between TFF3 expression with survival in 74 glioma patients.

Expression level of TFF3 was investigated in 92 glioma specimens and five normal brain tissue specimens by using immunohistochemistry. Strong TFF3 staining signal was identified under microscopy. In addition, no positive signal was found in normal brain tissue specimens. The intensity of positive TFF3 immunoreactivity was correlated with WHO grade (Figure 1A). Compared with Grade I/II specimens, TFF3 significantly elevated in Grade III and IV samples (*P*<0.001, Figure 1B). Notably, TFF3 expression level correlates with the malignancy of tumor that scored by tumor grade not correlated with patient age, sex, tumor size, tumor location, severity of edema, or presence of cystic change (*P*>0.05 each, Table 1).

**Table 1:** Correlations of TFF3 expression with preoperative and postoperative clinicopathological feature in glioma patients

	Variable	No. of cases	TFF3 expression			*p* Value
			1	2	3	
Age	< 60 years	63	15	21	27	0.735
	≥ 60 years	29	9	8	12	
Sex	Male	41	12	15	14	0.354
	Female	51	12	14	25	
Tumor size	< 4.5cm	48	13	15	20	0.974
	≥ 4.5cm	44	11	14	19	
Location	Non-eloquent	45	10	17	18	0.424
	Eloquent	47	14	12	21	
Edema	None to mild	42	10	12	20	0.649
	Moderate to severe	50	14	17	19	
Cystic change	Absent	40	11	10	19	0.486
	Present	52	13	19	20	
WHO grade	I & II	23	12	8	3	<0.001
	III	30	10	12	8	
	IV	39	2	9	28	

To further confirm these observations, the expression of TFF3 in glioma and normal brain tissues was verified at both the protein and mRNA levels. The results showed that TFF3 was also higher in glioma than in normal brain tissues and was higher in high-grade (WHO grades III and IV) than low-grade (WHO grades I and II) glioma samples (Figure 1C). The levels of TFF3 mRNA were significantly augmented in high-grade than low-grade gliomas (*P*<0.01, Figure 1D). Then, we measured the TFF3 mRNA expression in five malignant glioma cell lines (U87, U373, U251 T98G and LN229) and five normal brains as control. TFF3 mRNA expression also increased in glioma cell lines (*P*=0.003, Figure 1E). In addition, we also found glioma patients with low TFF3 expression (staining intensity score 1 and 2) have a prolonged median survival time compared with those with high TFF3 expression (staining intensity score 3) (1503 days vs. 563 days, Log-rank test, ***P<0.001, Figure 1F)

### TFF3 promotes cell proliferation, migration, invasion and suppresses apoptosis of glioma cell lines *in vitro*

**Figure 2:**
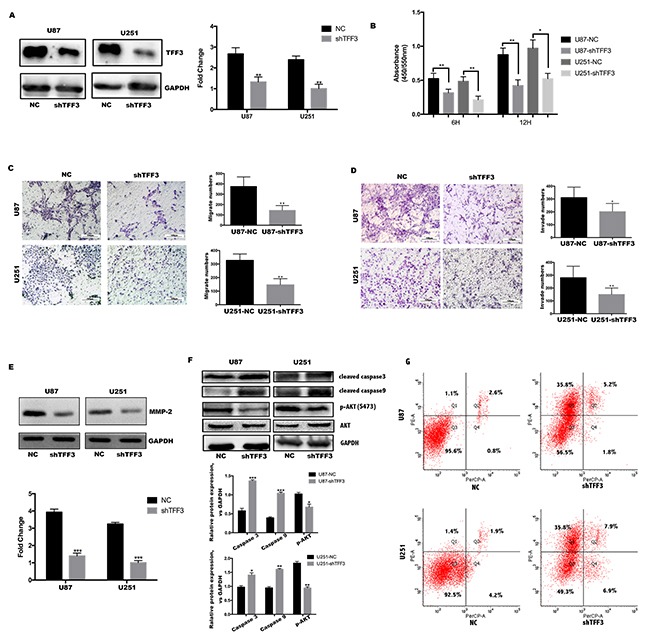
TFF3 promotes U87&U251 proliferation, migration, invasion and suppresses apoptosis **(A)** Western blotting analysis of TFF3 protein in the U87 and U251 cell lines after stable knockdown by the lentivirus. **(B)** U87 and U251 cells proliferation determined by 6 and 12 hours BrdU pulse assay (mean+SD, n = 3 biologically independent samples per group, one representative experiment shown, and the experiment was repeated 3 times, *P<0.05, **P<0.01). **(C, D)** Transwell migration and invasion assays of human glioma cell lines U87 and U251. Representative images are shown. Cells in five random fields of view were counted and expressed as the average number of cells per field of view(*P<0.05, **P<0.01). **(E)** Western blot analysis to determine the regulation of invasion-related protein MMP2 by TFF3 in U87 and U251 cells. Data are shown as mean+SD (n=3), ***P<0.001. **(F)** Western blot analysis shows that TFF3 knockdown increases the cleaved caspase3 and caspase9, as well as decreases p-AKT(S473). Data are shown as mean+SD (n=3), *P<0.05, **P<0.01, ***P<0.001. **(G)** Flow cytometry analysis shows that U87 and U251 increase rate of apoptosis after TFF3 knockdown, first quadrant indicates early apoptosis.

As cell proliferation and migration are major process during tumor progression. We tested the hypothesis that TFF3 regulating glioma cell proliferation, migration and invasion. First, U87 and U251 cell lines were transfected with TFF3 knockdown lentivirus and selected by puromycin to generate stable knockdown cell lines. The protein levels of TFF3 in U87-shTFF3 and U251-shTFF3 are lower than those in the NC groups (Figure 2A). Then, these cell lines were subjected to BrdU incorporation assays. Our data indicated that the cell proliferation were lower in the shTFF3 groups than the NC groups (**P<0.01, Figure 2B). Subsequently, in migration and invasion assays, TFF3 knockdown reduced cell motility and invasive ability (*P<0.05, **P<0.01) (Figure 2C-2D). More than simple locomotion, invasion ability of a given cell requires its breakthrough of the extracellular matrix. To explore the mechanism whereby TFF3 regulates glioma cell motility, we measured expression of the invasion- associated molecules MMP-2, finding that their protein levels significantly decreased after TFF3 knockdown in U87 and U251 cells (Figure 2E).

To explore the underlying mechanisms of TFF3 in the processes of cell apoptosis, we next investigated the functional role of the Akt signaling pathway. Akt, a key regulatory factor, is responsible for maintaining cell survival. To this end, the expression of phosphorylated Akt(S473), cleaved caspase-3 and cleaved caspase-9 were measured in different TFF3 knockdown glioma cell lines. According to the Western blotting results, both cleaved caspase-3 and cleaved caspase-9 levels were elevated in the TFF3-knockdown cell lines compared with the NC groups. In addition, TFF3 knockdown resulted in a decrease in protein expression of phosphorylated Akt (Figure 2F). Then 7AAD and PE were stained and analyzed by flow cytometry. After TFF3 knockdown, the rate of apoptosis significantly increased in both U87 and U251 cell lines (Figure 2G). Putting all data together, we conclude that Akt/Caspase signaling pathway involves in TFF3 protection against apoptosis in glioma.

### HIF-1α expression correlates with TFF3 in human glioma tissues

**Figure 3:**
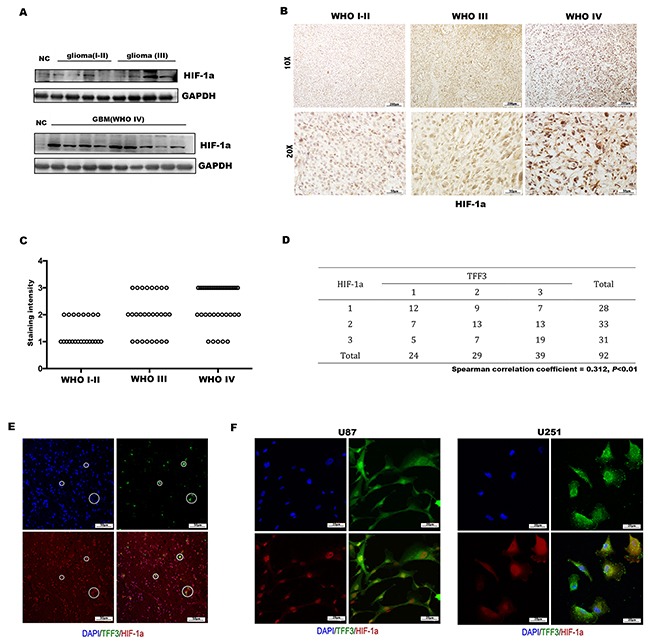
Expression of HIF-1α in glioma and correlation with TFF3 **(A)** Western blotting analysis showing that the overall expression of HIF-1α tended to increase along the sequence from NC (normal brain) to low-grade glioma (WHO grades I-II), and then to high-grade glioma (WHO grades III and IV). **(B)** Immunohistochemistry with anti-HIF-1α antibody on human glioma tissue samples showing nucleus positivity, one representative picture of each grade shown. **(C)** HIF-1α expression, represented by the staining intensity from WHO grade I-II to grade IV, showing statistically significant differences between grade IV and grade I-II, grade III (*P*<0.001, n=92). **(D)** Correlation of TFF3 and HIF-1α in 92 human glioma sections (Spearman correlation coefficient = 0.312, *P*<0.01). **(E, F)** Human tumor sections, U87 and U251 cells were stained with anti-TFF3 and anti-HIF-1α antibodies.

It has been reported that TFF3 regulates the expression of VEGF and HIF-1α induced by hypoxia, in gastric cancer cell lines [24]. However, the regulation between TFF3 and HIF-1α has not yet been elucidated in glioma. To this end, we first detected the protein level of HIF-1α in glioma tissues and normal brain tissues. As expected, HIF-1α was dramatically overexpressed in high-grade glioma tissues as demonstrated by Western blotting analysis (Figure 3A). To further confirm these observations, immunohistochemistry was preformed using 92 glioma tissues. Similar to the results of TFF3 expression in different grade glioma samples (Figure 1A), higher HIF-1α expression was found in high-grade (WHO grades III and IV) than low-grade (WHO grades I and II) glioma tissues (Figure 3B), showing a statistically significant difference between these groups (*P*<0.001, Figure 3C). The correlation between TFF3 and HIF-1α was analyzed using chi-square by putting numbers of cases of each staining intensity score into 3*3 crosstab. The expression level between TFF3 and t HIF-1α have a significantly positive correlation (Spearman correlation coefficient = 0.312, *P*<0.01, Figure 3D). To validate the interaction between TFF3 and HIF-1α in glioma, colocalization was assessed in both glioma tissues and the glioma cell lines. The results showed that TFF3 was colocalized with HIF-1α in the nucleus (Figure 3E-3F).

### HIF-1α promotes the proliferation, migration and invasion of glioma cell lines *in vitro*

**Figure 4:**
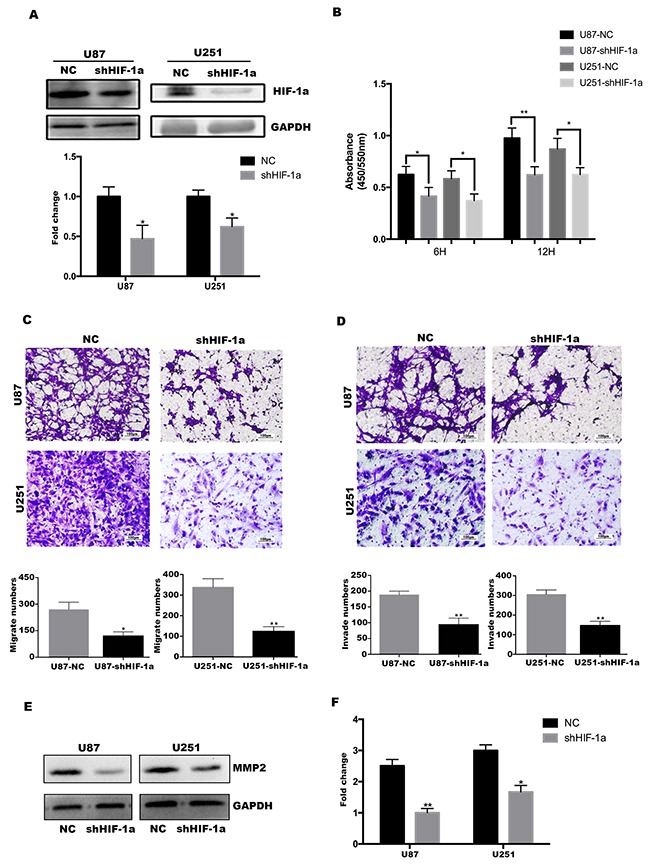
HIF-1α promotes U87&U251 proliferation, migration and invasion **(A)** Western blotting analysis of HIF-1α protein in the U87 and U251 cell lines after stable knockdown by the lentivirus, *P<0.05. **(B)** U87 and U251 proliferation determined by 6 and 12 hours BrdU pulse assay (mean+SD, n = 3 biologically independent samples per group, one representative experiment shown, and the experiment was repeated 3 times, *P<0.05, **P<0.01). **(C, D)** Transwell migration and invasion assays of human glioma cell lines U87 and U251. Representative images are shown. Cells in five random fields of view were counted and expressed as the average number of cells per field of view (*P<0.05, **P<0.01). **(E, F)** Western blot analysis to determine the regulation of invasion-related protein MMP2 by HIF-1α in U87 and U251 cells. Data are shown as mean+SD (n=3), *P<0.05, **P<0.01.

Given the interaction between TFF3 and HIF-1α, the function of HIF-1α should be investigated. First, stable HIF-1α knockdown U87 and U251 cell lines were established (Figure 4A). Similar with TFF3, both U87-shHIF-1α and U251-shHIF-1α exhibit a lower rate of proliferation, migration and invasion (Figure 4B-4D). MMP-2 protein expression also decreased in U87 and U251 cells after knockdown HIF-1α (Figure 4E-4F).

### HIF-1α is critical for TFF3 mediated function in glioma cells

**Figure 5:**
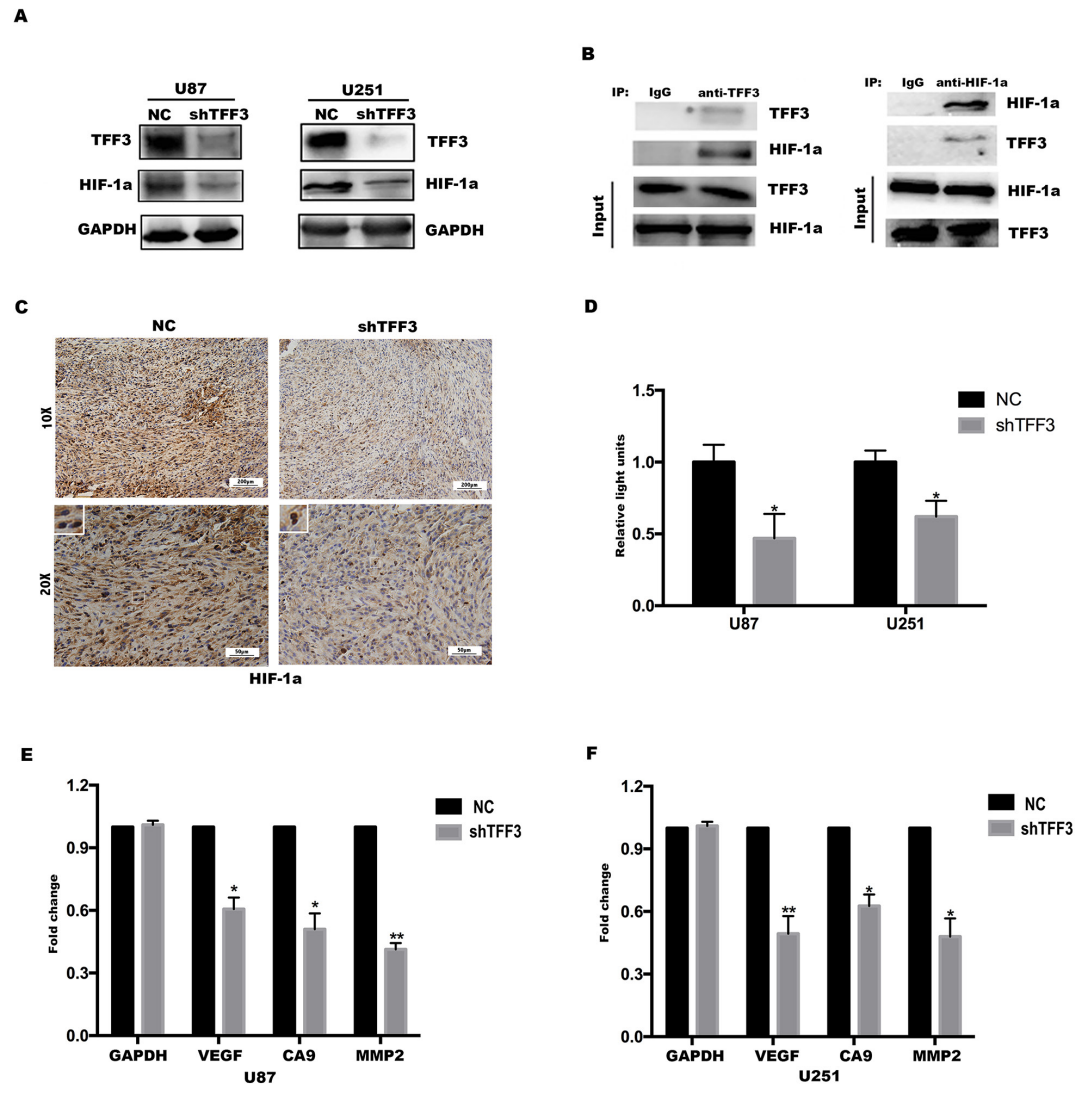
HIF-1α is critical for TFF3 mediated function in glioma cells **(A)** Western blotting showing that the levels of HIF-1α protein expression were significantly lower in TFF3 knockdowned cell lines (**P<0.01). **(B)** TFF3 stable-overexpressed U87 cell lysates were immunoprecipitated with anti-TFF3 or anti-HIF-1α antibody and subjected to immunoblot analysis. **(C)** Immunohistochemistry with anti-HIF-1α antibody on nude mice tumor samples. **(D)** Stable TFF3 knockdown U87 and U251 cells were transfected with a HRE luciferase vector, and HRE transcriptional activity was assayed (*P<0.05). **(E, F)** VEGF, CA9 and MMP2 mRNA were determined by qRT-PCR (*P<0.05, **P<0.01).

Since TFF3 was cooperated with HIF-1α to promote the tumorigenesis of glioma, we tried to investigate whether TFF3 regulates the expression of HIF-1α, Western blotting analysis was performed. As shown in Figure 5A, the protein level of HIF-1α were reduced by knockdown of TFF3 compared with the NC groups. To understand why knockdown of TFF3 cause the decrease of HIF-1α, co-IP analysis was carried out to detect the interaction between TFF3 and HIF-1α. The result suggested that TFF3 could bind to HIF-1α in glioma cell lines (Figure 5B). Therefore, these results strongly suggest the involvement of TFF3 in regulating HIF-1α expression in gliomas. But, the molecular regulatory mechanisms of TFF3 and HIF-1α require further exploration.

Next, a xenograft mouse model was established to investigate whether TFF3 regulates the expression of HIF-1α *in vivo*. U87-shTFF3 and U87-NC cells were injected subcutaneously. 4 weeks after injection, the mice were sacrificed and the tumors were dissected out, followed by fixation with 10% formalin and embedment in paraffin. The expression of HIF-1α was determined by immunohistochemistry assay. As shown in Figure 5C, the signal intensity of HIF-1α was significantly lower in shTFF3 group than in NC group (one representative picture shown, *P*<0.001, n=8) and this result was consistent with the data *in vitro*.

HIF-1α is an oxygen-dependent transcriptional factor, which plays crucial roles in the tumor progression. The target genes of HIF-1α are especially related to cell proliferation/survival, apoptosis and glucose/iron metabolism [25]. Therefore, we presume that TFF3 regulates the transcriptional activity of HIF-1α and thus promotes glioma cells proliferation, migration and invasion. HIF-1α transcriptional activity was measured using a hypoxia response element (HRE) luciferase reporter. TFF3 knockdown led to a decrease in HRE activity (Figure 5D). This shows that TFF3 promotes HIF-1α transcriptional activity, which was also reflected in the decrease in expression of the HIF-1α target genes VEGF, MMP2 and CA9 in response to TFF3 knockdown (Figure 5E-5F).

### Silencing TFF3 delays tumor progression and prolongs the survival of mice bearing intracranial xenografts

**Figure 6:**
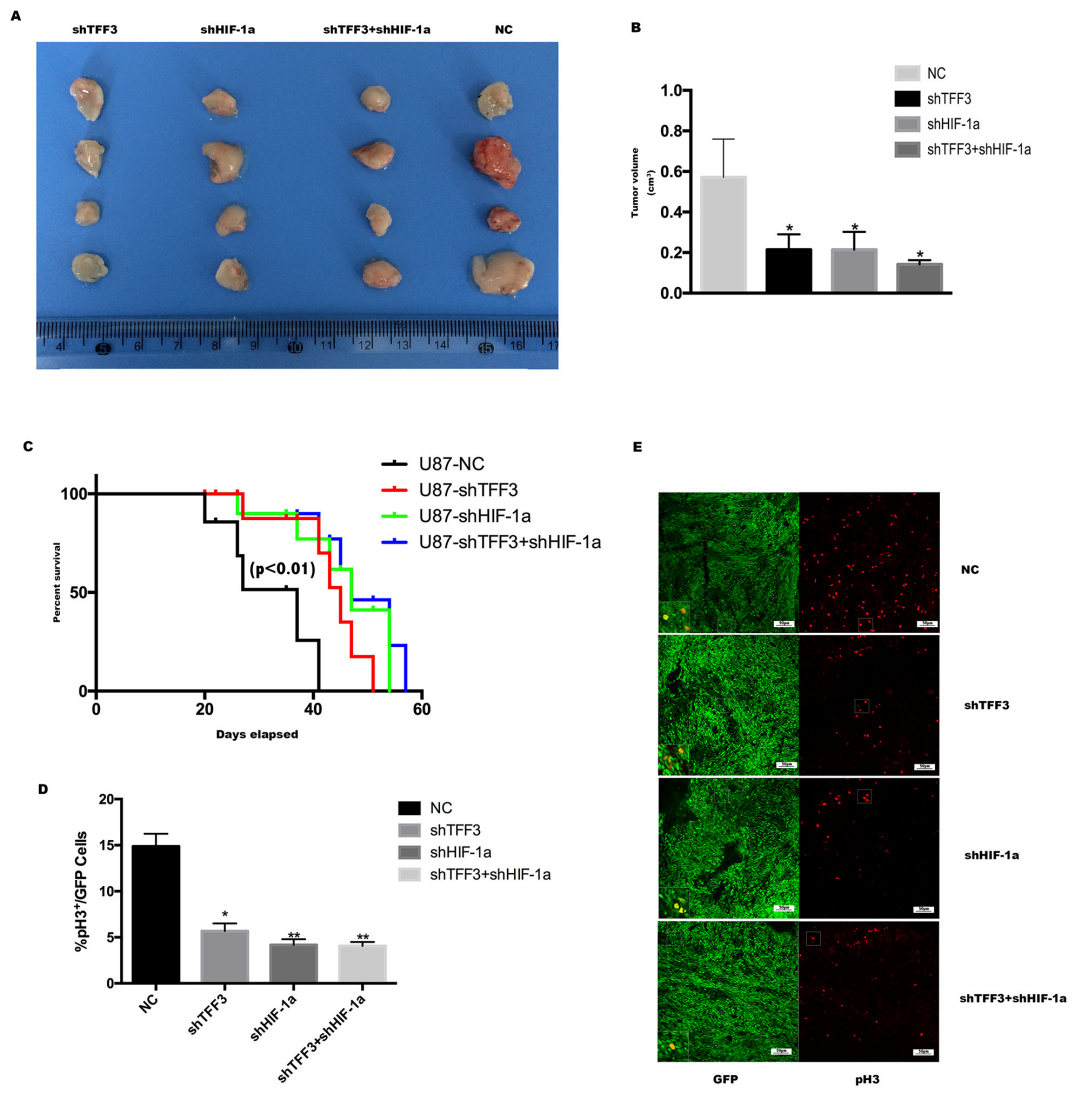
Silencing TFF3 delays tumor progression and prolongs the survival of mice bearing intracranial xenografts **(A)** U87 cells were transfected with siTFF3, siHIF-1α and NC lentivirus and were injected subcutaneously caudal to the forelimbs in 4- to 6-week-old BALB/c nu/nu mice. **(B)** Subcutaneous tumors were resected and exhibited a significant difference in tumor volume (*P<0.05). **(C)** Kaplan-Meier survival curves of mice bearing intracranial xenografts derived from four stable transfected U87 cell lines mentioned above (*P*<0.01). **(D)** Percentage of phospho-Histone H3 positive cells in all GFP positive cells in intracranial xenograft mice (n=20, 5 mice in each group, *P<0.05, **P<0.01). **(E)** Representative pictures are shown. Green is GFP staining and red is pH3 staining.

To further characterize the impact of TFF3 *in vivo*, subcutaneous tumors were generated with U87-shTFF3, U87-shHIF-1α, U87-shTFF3/shHIF-1α and NC cells in immunocompromised mice (NOD-SCID) (Figure 6A). A significant difference in tumor growth at the end of the experiment was detected between shTFF3/shHIF-1α/shTFF3&shHIF-1α and NC groups. However, there is no significant difference among the three treated groups (Figure 6B, *P<0.05). Then, intracranial implantation of these cells significantly increased the mean survival of tumor-bearing mice compared with NC group (Figure 6C, 45, 47 and 47 vs. 35 days, Log-rank test, *P*=0.007). Then, tumors were cryosectioned and stained with GFP and phospho-histone H3 (Ser 10) to measure the proliferation rate of tumor cells. Similar with *in vitro* study, the proliferation rate of tumor cells decreased in U87-shTFF3 and U87-shHIF-1α (Figure 6D-6E).

## DISCUSSION

TFF3, a member of the trefoil peptide family, is conserved among species and has a trefoil domain and C-terminal dimerization domain [26]. TFF3 is secreted to the intestinal surface by goblet cells and plays an important role in the reconstitution of the mucosal barrier and maintaining mucosal integrity [27, 28]. In gastrointestinal (GI) cancer cell lines, TFF3 was found to inhibit cell adhesion and promote cell invasion [29, 30]. In breast tumors, TFF3 promotes angiogenesis [31, 32]. It has been reported that the combination of serum TFF3 and pepsinogen testing could be a biomarker for early detection of gastric cancer [33–35]. However, a comprehensive understanding of the mechanistic role of TFF3 in tumorigenesis remains unclear and deserves further investigation. In this study, we focused on the role of TFF3 in glioma development and identified TFF3 as an oncoprotein in glioma.

It was observed that TFF3 expression was accompanied by an increase in HIF-1α protein. In addition, TFF3 was physically associated with HIF-1α and colocalization in nucleus. HIF-1α is a critical transcription factor implemental in regulating diverse cellular responses to hypoxia, inducing expression of multiple target genes that drives aberrant proliferation and invasion during tumor progression [36]. Here, we show a hypoxia-independent mechanism of HIF-1α induction in glioma, in which TFF3 cooperates with HIF-1α by upregulating its expression and transcriptional activity. Thus, elevated HIF-1α induces the elevation of multiple target genes, e.g. VEGF, CA9 and MMP2, eventually resulting in aberrant proliferation, invasion and tumor progression.

In previous studies, TFF3 is induced and parallel to HIF-1α under hypoxic conditions [37, 24]. However, we surprisingly find that TFF3 directs accumulation of HIF-1α even under normoxic conditions. Normoxic induction of HIF-1α by human epidermal growth factor-like receptor 2 (HER2) has been previously reported [38]. HER2 expression correlates with the breast cancer progression and is an important diagnostic biomarker and therapeutic target for breast cancer, but is overexpressed in only 10-15% of GBM tumors [39]. Likewise, TFF3 expression correlates with the WHO grade of glioma (Figure 1), regulating the expression and transcriptional activity of HIF-1α under normoxic conditions (Figure 5). In order to identify the function of TFF3 and HIF-1α playing in glioma, we constructed U87 and U251 TFF3-knockdown/ HIF-1α-knockdown stable cell lines. Stable knockdown of TFF3 significantly reduced glioma cells proliferation and invasion *in vitro* (Figure 2) and suppressed the tumor volumes *in vivo* (Figure 6). Furthermore, we tried to disclose the mechanism how TFF3 involved in apoptosis. In this assay, knockdown of TFF3 upregulated cleaved Caspase3/9 expression and suppressed *p*-AKT expression and thus promoted tumor cells apoptosis, which suggested that TFF3 blocked apoptosis in glioma cell lines (Figure 2). Importantly, patients with glioma expressing low levels of TFF3 have significantly prolonged survival time compared to those expressing high levels of this gene. Similar phenotype was observed in intracranial xenografts mice (Figure 6). Thus, inhibition of TFF3 may offer a selective therapeutic strategy for targeting expression of HIF-1α in gliomas.

In conclusion, TFF3 drives aberrant glioma cells proliferation and invasion *in vivo* and *in vitro* through a hypoxia-independent HIF-1α induction. Parallel with HIF-1α, the expression of TFF3 was correlated with the WHO grade of gliomas. As a corollary, although the mechanism of TFF3-HIF-1α interaction needs further investigation, inhibition of TFF3 might offer a novel strategy to target HIF-1α and retard glioma progression.

## MATERIALS AND METHODS

### Cell lines and cell culture

Human glioma cell lines U87, U373, T98G, LN229 and U251 were purchased from the American Type Culture Collection (Manassas, VA, USA). All cells were cultured in DMEM (Hyclone, Thermo Fisher Scientific, Waltham, MA, USA) supplemented with 10% fetal bovine serum (FBS; Hyclone) and 100 U/mL penicillin/streptomycin (Gibco) at 37 °C. The recombinant lentivirus vectors were bought from GenePharma (Shanghai, China). U87 and U251 cells were transfected with the lentivirus respectively as follows: pLKD-CMV-GFP-puro-U6-shTFF3, pLKD-CMV-GFP-puro-U6-shHIF-1α and pLKD-CMV-GFP-puro-U6-NC for 72 h, and 2.5μg/ml puromycin was added to the medium, and cells were passaged for over ten generations. Finally, six purified individual clones were selected: U87-shTFF3, U87-shHIF-1α, U87-NC, U251-shTFF3, U251-shHIF-1α and U251-NC.

### Clinical glioma samples

Fresh human glioma samples collected from the neurosurgery department of China Medical University from 40 adult glioma patients had been freshly resected during surgery. Five normal brain tissue samples came from traumatic intracranial surgical decompression. All samples were immediately frozen in liquid nitrogen and stored at -80 °C. Then, total RNA from these tissues was isolated. The rest of the tissues were fixed with formalin and embedded in paraffin. Another 52 cases of paraffin-embedded tissue were collected from Dalian Medical University in China. None of patients had received chemotherapy, immunotherapy or radiotherapy prior to the surgery. We followed all the patients after surgery and collected dead point of 74 cases. All patients gave written informed consent according to a study protocol that was approved by the Ethics Committee of China Medical University and the Ethics Committee of Dalian Medical University.

### Quantitative RT-PCR

Total RNA from tissues was isolated using a MiniBEST Universal RNA Extraction Kit (TaKaRa) according to previous study [40]. The expression of TFF3, VEGF, CA9 and MMP2 was determined using a One Step SYBR^®^ PrimeScript RT-PCR Kit (TaKaRa) and normalized to the expression of glyceraldehyde 3-phosphate dehydrogenase (GAPDH). The following primers were used: TFF3 mRNA forward: 5′- TGAACTGACCTCTCCCCTTT -3′, reverse: 5′- CTGCTCTGGATTGTTTGCTTG -3′, VEGF mRNA forward: 5’- GCTACTGCCATCCAATCGAGAC-3’, reverse: 5’- CTATGTGCTGGCCTTGGTGA G-3’, CA9 mRNA forward: 5’- ACCTGGTGACTC TCGGCTACAG-3’, reverse: 5’- CAG CCAGGCAGGAA TTCAGC-3’, MMP2 mRNA forward: 5’- CCCACACTGGGCCCTGTCACT-3’, reverse: 5’- TGGGCTTGTCACGTGGCGTC-3’, GAPDH mRNA forward: 5′- CGGAGTC AACGGATTTGGTCGTAT -3′, reverse: 5′-AGCCTTCTCCATGGTGGTGAAGAC -3′ (TaKaRa). The comparative Ct (2^-ΔΔCt^) method was used [41].

### Western blot and immunoprecipitation assay

Total cellular and tissue protein was extracted using RIPA lysis buffer containing protease and phosphatase inhibitors (Thermo Fisher, USA). Samples were incubated for 20 min on ice after sonication with an ultrasonic cell crusher and then centrifuged for 20 min at 13,000 rpm at 4°C. Protein concentrations were determined using the Pierce BCA protein assay kit (Thermo Fisher, USA). Immunoprecipitation was performed using Pierce Protein A/G Magnetic IP/Co-IP Kit (Thermo Fisher, USA) following standard protocol. Lysate samples (40 μg) were separated with 12% SDS-PAGE and transferred onto PVDF membranes (Millipore, USA). Membranes were incubated with primary antibodies overnight at 4°C. Membranes were washed with TBST and incubated for 2 h with HRP-conjugated anti-rabbit secondary antibodies (1:5000; CST, USA), followed by detection and visualization using ECL Western blotting detection reagents (Pierce, Thermo Fisher, USA). The primary antibodies used were anti-TFF3 (1:1000; ab108599, Abcam, USA), anti-HIF-1α (1:200; ab1; Abcam, USA), anti-MMP2 (1:1000; ab86607; Abcam, USA), anti-AKT1 (phospho S473) (1:1000; ab81283; Abcam, USA), anti-cleaved caspase-3 (1:1000; #9664; CST, USA), anti-cleaved caspase-9 (1:1000; #7237; CST, USA)

### Bromodeoxyuridine assay

The incorporation of bromodeoxyuridine (BrdU) into the DNA of prolifertating U87 and U251 cells were measured according to the manufacture's protocol of BrdU Cell Proliferation ELISA Kit (ab126556, Abcam, USA). Briefly, the cells were cultured in 96-well plate at a density of 3,000 cells per well in growth media. After 24h, the cells were labeled using 1X BrdU at pulse of 6h and 12h. After incubation, the culture media was removed and DNA was denatured by adding Fixing Solution. Next, the cells were incubated with the primary anti-BrdU antibody for 1h at room temperature. After washing the first antibody, Peroxidase Goat Anti-Mouse IgG Conjugate was added and incubate for 30 minutes at room temperature. Last, the substrate solution was added and the reaction product was quantified by measuring the absorbance at 450/550nm.

### Transwell invasion and migration assay

Eight μm pore size transwell filters (Corning, USA) were precoated with/without Corning Matrigel matrix in a 24-well plate. Stably transfected U87 and U251 cells (4×10^4^) in 100 μL of serum-free medium were seeded into the upper chamber. The lower chamber was filled with 500 μL of medium supplemented with 5% FBS. After 24 h of incubation, the cells in the upper chamber were removed by a cotton tip. The cells in the lower chamber were stained with crystal violet. Invading cells were counted in five randomly chosen fields under a microscope.

### Xenograft mouse model

U87 cells were stably transfected with lentivirus shTFF3, shHIF-1α or NC. Cells (10^6^) were injected subcutaneously just caudal to the forelimbs in 4- to 6-week-old BALB/c *nu/nu* mice (Charles River, Beijing, China). Tumor diameters were measured with calipers at 7-day intervals, and volumes were calculated from four mice per data point (mm^3^ = width^2^ × length /2). For the intracranial inoculation, mice were anesthetized and a Hamilton syringe was used to inject 5×10^5^ stable transfected cells through a 3-mm hole to the right of bregma, at a depth of 2.5 mm. The surgical zone was flushed with sterile saline, and the hole sealed with bone wax, and the skin over the injection site sutured. All animals were monitored daily and sacrificed at the end of the experiment. Then the whole brain was collected and fixed in 4% paraformaldehyde and cryoprotected in 30% sucrose. The brain was cryosectioned sagittally and stained with anti-GFP (1:200; CST, 2956S, USA) and phospho-Histone H3 (Ser 10) (1:200, Millipore, 06-570, USA). The experimental protocol was approved by the Dalian Medical University Animal Care & Use Committee, and all animals were housed in the Laboratory Animal Center of Dalian Medical University.

### Transfections and dual luciferase assay

U87 cells were transferred to 48-well plates (2 × 10^4^cells/well) one day before transfection. Cells were transfected with 175 ng HRE luciferase reporter plasmids (Promega). The plasmids were premixed with transfection reagent Lipo 2000 (Invitrogen). The next day, cells were harvested and Dual Luciferase reporter assay kit (Promega) was used for measurements.

### Immunofluorescence

Human biopsy specimens from subjects with several tumor types, including astrocytoma, oligodendroglioma, ependymoma or GBM, were de-paraffinized, rehydrated, and subjected to antigen retrieval in low pH Target Retrieval Solution (Dako) at 95°C for 20 min. Sections were blocked with 5% BSA for 4 h at room temperature, incubated with anti-TFF3 (1:100, Abcam, USA) and anti-HIF-1α (1:100, Abcam, USA) antibodies overnight at 4°C. Cultured cells were fixed with 4% paraformaldehyde for 20 min and incubated with anti-TFF3 (1:100, Abcam, USA) and anti-HIF-1α (1:100, Abcam, USA) antibodies overnight at 4°C. Sections were stained with Alexa Fluor 488- and 647-conjugated secondary IgG (1:500, Abcam) for 1 h at room temperature. Images were acquired with an FV1000 Laser Scanning Confocal Microscope (Olympus, Japan).

### Immunohistochemistry

Histological sections of the tumor tissue were embedding in paraffin. Sections of 4 μm were cut and dried, deparaffinized, and rehydrated before antigen retrieval in low pH Target Retrieval Solution (Dako) at 95°C for 20 min. Sections were blocked with 5% BSA for 2 h at room temperature and incubated with anti-TFF3 (1:100, Abcam, USA) or anti-HIF-1α (1:100, Abcam, USA) at 4°C overnight. Dako REAL EnVision Detection System (Dako, Denmark) was used according to the manufacturer's protocol. Sections were then dehydrated in ascending grades of methanol, cleared with xylene, and covered with a coverslip.

Cells scored positive for TFF3 and HIF-1α immunostaining had a brown color in the nucleus and/or cytoplasm. TFF3 and HIF-1α expression was classified semi-quantitatively by taking into account the intensity of staining. Tumor staining intensity was rated as follows: 1 point, weak intensity; 2 points, moderate intensity; 3 points, strong intensity.

### Flow cytometry

The apoptosis of the cells was assayed by staining with Annexin V-PE (BD Biosciences, USA) and detected by flow cytometry. Cells were collected and washed with ice-cold PBS and stained with 100μL binding buffer containing 5μL Annexin V-PE and 5μL 7-AAD at room temperature in the dark for 20min. For each experiment, 10,000 cells were analyzed using the BD FACSCalibur. All the experiments were performed in triplicate.

### Statistical analysis

All experiments were repeated independently at least 3 times, and the data were expressed as the mean+SD/S.E.M using SPSS. The significance of the differences between groups was determined using a 2-tailed Student's t test, one-way ANOVA or chi-square test. The survival analysis in glioma patients and intracranial xenograft mice were modeled with Kaplan-Meier method. *P* < 0.05 was considered a statistically significant difference.
